# Collaborating on Early Detection of Frailty; a Multifaceted Challenge

**DOI:** 10.5334/ijic.4176

**Published:** 2019-04-23

**Authors:** Yvette Buist, Mieke Rijken, Lidwien Lemmens, Caroline Baan, Simone de Bruin

**Affiliations:** 1 National Institute for Public Health and the Environment, Centre for Nutrition, Prevention and Health Services, Bilthoven, NL; 2 Netherlands institute for health services research, Utrecht, NL

**Keywords:** early detection, preventive elderly care, collaboration, integrated care, qualitative study, frailty

## Abstract

**Introduction:**

In several countries, initiatives to detect frailty among older citizens at an early stage are being implemented to enable proactive intervention and, consequently, to support independent living for as long as possible. Alignment and collaboration between the various actors are crucial. We aimed to provide insight in factors that impede or facilitate collaboration at a local level as perceived by the different actors and we explore their experiences.

**Methods:**

Semi-structured interviews were conducted with 37 representatives of three groups active in proactive elderly care in the Netherlands: (i) commissioners, (ii) service providers, and (iii) other stakeholders (e.g. public health advisors, academics). The Framework Method was used to analyse data.

**Results:**

Interviewees perceived many factors hampering or facilitating collaboration. Overall, the factors mentioned were quite similar for the different groups. Facilitators and barriers were related to culture and professionals (e.g. knowledge of early detection approaches, mutual trust), organizations (e.g. shared vision or patient information system) and context (e.g. financing).

**Discussion and conclusion:**

Collaborating on early detection appears to be a multifaceted challenge. However, as different stakeholders hold similar views, there seem to be several starting-points to improve collaboration. First steps shall include getting to know each other and developing a shared vision on early detection.

## Introduction

Today, countries all over the world face problems associated with an increasingly ageing population, which brings about major challenges for their health systems and societies. One of these challenges is the increase of older people who have a frail health status or are at a high risk of developing frailty [1]. There is no general consensus on the definition of frailty [2,3]. Frailty can concern physical frailty as well as social and psychological frailty. Prevalence rates among older (65+) adults living in the community have been found to range from 4% to 17% in studies operationalising frailty as a purely physical condition [2], and from 2% to 60% [2,4] in studies applying a broader definition of frailty which also take cognitive, psychological and social aspects into account.

A frail status implies a high risk of adverse outcomes, such as falls, dependency, loneliness, hospitalization and premature mortality [5,6,7,8]. Frailty is, as such, a serious threat for older individuals’ wellbeing as well as for high public expenditures. Several studies show that recognizing (potential) frailty at an early stage provides options for proactive care, and could as such delay or reduce functional decline, prevent hospital admissions, and maintain wellbeing and independent living for as long as possible [9,10,11,12]. Apart from preventing deterioration, Dury et al. [13] argue that early detection of frailty is also important to identify and strengthen older persons’ resources to counterbalance their frail health status.

The attention of policymakers and care authorities for the potential benefits of early detection of frailty is growing. This is reflected in the development of many initiatives that include activities regarding early detection, such as the identification of older people with frailty or frailty related symptoms in primary care, geriatric care assessments, population-based multidimensional assessments by public health organizations and, preventive home visits by voluntary advisors or care professionals [9, 14,15,16,17]. These initiatives are also aimed at different target populations, e.g. frail older people in primary care or hospitalized older people. Home visits by professionals or informal carers are often part of early detection initiatives and usually include a needs assessment [16,17,18]. The setting and professionals and/or informal carers involved in early detection will mostly determine the focus of the initiative. This implies that some initiatives mainly focus on physical health problems, whereas others focus more on psychological, social or environmental domains of life. This reflects the broad spectrum of definitions of frailty. Hence, the focus of the needs assessment and follow-up activities may differ. Despite these differences in focus, earlier studies suggest that there is often duplication between different services [17,19].

In the Netherlands, there is a broad legal basis for early detection of (a high risk of) frailty, as it is incorporated in several laws, i.e., the Health Insurance Act, the Social Support Act and the Public Health Act (see Table 1). Given that these laws provide different approaches to early detection and vary in their levels of accountability and execution, they carry the risk that early detection initiatives at a local level lack coordination and alignment, which may negatively impact their quality and efficiency. Therefore, it is essential that all actors involved in early detection initiatives at a local level know of each other and collaborate as much as possible. Previous studies, however, provide indications that this is not common practice yet [17].

**Table 1:** Legal framework, settings and care providers involved in early detection initiatives in The Netherlands.

Legal basis	Setting	Service provider

Health Insurance Act: Objectives and budget set by central government. Executed by private healthcare insurance companies, which contract care providers.	General practice	General practitioner Practice nurse Elderly care specialist (consulting role)
	Home care organization	Community nurse
	Hospital	Geriatrician Transfer nurse Nurse specialist/nurse practitioner
Social Support Act 2015: Objectives and budget set by central government. Executed by municipalities, which contract care providers.	Community service, Social care organization	Social support counsellor Social worker
No specific legal basis.	Senior citizens organization	Volunteer elderly advisor
	Social network, neighbourhood facilities	Family, friends, neighbours, pastor, etc.
Public Health Act: Early detection of frail individuals does not take place, but a population approach is used in which municipalities focus on the broad population of the elderly in their municipality, for example by monitoring vulnerability, well-being and safety.	Municipality, public health services	Municipality policymakers Advisors on Public Health

There are several factors that impede collaboration between health and social care services, such as competing organizational visions or different professional visions and values, separate management structures and inadequate resources [20,21]. Factors that promote inter-sectoral collaboration have also been found, such as strong leadership, appropriate professional support and bundled payment. It seems likely that such factors also play a role in inter-sectoral collaboration on early detection. However, as frailty is a complex phenomenon and service providers from health and social care services may hold different views of frailty (either being a predominantly physical or a multidimensional condition), collaboration on early detection may be affected by specific factors. Up until now, insight is lacking in which factors are particularly key for successful collaboration in early detection initiatives. In addition, as there is still little knowledge on effective early detection approaches and consensus is lacking, service providers at a local level may have different opinions and apply different methodologies to detect frailty among their target populations. Also, as mentioned above, these service providers act on early detection from different settings, which include differences in legal basis, overall aims and target populations of their services. The ambiguous nature of frailty, the heterogeneity of early detection approaches applied, as well as the different context of the service providers involved bring about additional challenges for alignment and collaboration. This may imply that the various stakeholders perceive different barriers and facilitators for collaboration on early detection. Therefore, the aims of this study were: 1. to provide insight in factors that impede or facilitate collaboration, according to actors involved in initiatives to detect frailty among older citizens, and 2. to gain insight in whether different groups of actors, such as commissioners, managers and professionals of care organisations, advisors, academics and representatives of organizations for older people, hold different views regarding these factors. These insights are important to be able to identify those factors that are most important for improving collaboration between professionals and agencies involved in early detection at a local level.

## Methods

### Study design and study sample

This qualitative study was conducted between April and August of 2017 in the Netherlands.

A broad range of actors in proactive elderly care was invited to participate in an interview. The sample included three groups:

1. Commissioners: municipality policymakers (n = 9) and representatives of health insurance companies (n = 3);
2. Service providers: managers from health and social care organizations (n = 9) and health and social care professionals (n = 8);
3. Other stakeholders: public health advisors from local (care) support centres (n = 4), academics (n = 2) and representatives of organizations for older people (n = 2).

Actors were recruited using purposeful sampling. Some actors were directly involved in a collaborative initiative on early detection. Others were aware of collaborative initiatives due to their position, e.g. advisor or researcher. Actors were selected: 1. from the researchers’ network; 2. from the literature on early detection studied during the preparation phase of this study, and 3. on the basis of meetings with actors and network meetings. The selected actors were sent an email including a description of the study and an invitation to participate. When the researcher received a positive response, time and place for the interview were determined.

A total of 18 interviews were conducted with 37 actors (21 female and 6 male). Twenty-seven actors were invited to participate, of whom 21 agreed to participate. Reasons for non-participation were non-response, a lack of time and change of position. The actors who agreed to participate were also asked to invite other colleagues to join them in the interview. This resulted in 16 actors who additionally joined the interviews. Four interviews took place with a mixed group of professionals. The other interviews were individual interviews or interviews with professionals with the same position. Per interview, one to four people participated. Prior to the interview, participants received information regarding the aims of the interview. The participants also received a written informed consent emphasizing voluntary participation and guaranteed anonymity. We interviewed professionals from eight different provinces throughout the Netherlands.

### Data collection

The interviews were guided by a semi-structured interview format. The interview guide was reviewed by an advisory committee consisting of three experts in the field of proactive care and some final adaptations were made according to their comments. During the interviews, two themes were addressed: 1. facilitators and barriers for collaboration and 2. alignment between collaborating actors and organizations. Since the context was different depending on the profession of the participants, there was a slight difference in focus of the questions asked. That is, health and social care actors were asked to relate to their experiences in practice, while public health advisors from local (care) support centres were asked to answer from a more overarching perspective. For example the participants who were directly involved in a collaborative initiative on early detection were asked how collaboration between the different actors involved in early detection was organized and what could be improved and participants with a rather visionary point of view on early detection were asked how collaboration between the different actors should be organized ideally. To verify whether similar facilitators and barriers were experienced or recognised by the actors, they were asked whether they were familiar with facilitator and barriers mentioned in preceding interviews. Most interviews took place face-to-face at the actors’ workplace. Three interviews were conducted by telephone. Interviews lasted between 33 and 87 minutes. The interviews were conducted by two experienced interviewers of our project team.

### Data analysis

All interviews were recorded and transcribed verbatim. The transcripts were checked for completeness by the researches who conducted the interviews. The interviews were analysed using the Framework Method for management and analyses of qualitative data [20]. The Framework Method is a method for structured content analyses of qualitative data [20].

In accordance with this framework, a coding scheme was developed by the interviewers based on the interview format (i.e. deductive approach) and on the topics discussed during the interviews (i.e. inductive approach). Two interviews were coded independently by two researchers of our project team. Then the coded items were compared, differences in coding were discussed, new codes were added (i.e. inductive approach) and an agreement was reached on how to continue coding. To use the richness of the data, new codes were added to the coding tree when items provided additional information to answer the research question. To minimize subjectivity all interviews were coded by one researcher and checked by another researcher of our project team. The qualitative analyses program MAXQDA 12 was used to code the transcripts. After coding the interviews, all coded data was examined by interpreting (recurring) items. Then drafts of the study findings were written and these findings were discussed by the authors. Additionally, a draft report on the study findings was sent to three actors in proactive elderly care (a municipality policymaker, an advisor from a regional support center and a representative of an organization for older people), of whom two had also participated in an interview, to validate our findings [22]. Through this process, it was established whether our findings were understandable and whether their viewpoints were adequately interpreted. Their feedback was used to refine findings further.

### Ethics statement

The Medical Research Ethics Committees United assessed the study proposal and concluded that the Medical Research Involving Human Subjects Act (WMO) does not apply to this study, and official approval was not required. All participants provided written informed consent.

## Results

Table 2 shows the factors that were mentioned by the participants to influence collaboration in the field of early detection. To classify the reported facilitators and barriers, we used the categories suggested by Cameron et al. [21], which organizes the factors that facilitate or hinder collaboration, at the following levels: organizational, cultural/professional and contextual. We mention the perspectives of the three different groups (i.e. commissioners, service providers and other stakeholders) and when relevant, we specify the subgroups within the three groups of participants.

**Table 2:** Facilitators or barriers mentioned at the organizational, cultural/professional and contextual level.

Groups	Commissioners		Service providers		Others		

Sub-groups	Municipalities policymakers (n = 9)	Representatives of health insurers (n = 3)	Managers from health and social care organizations (n = 9)	Health and social care professionals (n = 8)	Advisors from regional support centers (n = 4)	Academics (n = 2)	Representatives of organizations for older people (n = 2)

**Organizational level**							

Knowing each other’s tasks and responsibilities	X	X	X	X	X	X	
Shared vision on early detection	X		X	X	X		
Having seen the benefits of collaboration	X	X	X	X		X	
Time available	X		X	X	X	X	X
Shared patient information system			X	X		X	X
Organizational structure of municipalities	X	X	X	X		X	X
Dealing with fragmented funding	X		X				X
**Cultural and/or professional**							

Knowledge on early detection approaches		X	X	X			X
Knowledge on privacy legislation	X	X	X	X			X
Trust	X	X	X	X	X		
Leadership	X	X	X	X	X	X	X
**Contextual**							

Continuity of funding for collaboration	X		X	X	X	X	
Continuity of cooperation partners	X		X	X	X	X	

### Organizational level

Respondents from the three groups all noted that many professionals *do not know each other* and are not *aware of each other’s tasks and responsibilities*, which is a barrier for collaboration (see Table 2). According to the respondents, this is partly related to the size of the municipality in which health and social care professionals are active. Professionals working in small municipalities are more often familiar with each other’s field of work and expertise, while this is the case to a lesser extent in larger municipalities.

“It is important to know each other, to know what everyone is doing, to know about each other’s expertise, to trust each other, that there is a joint motivation, a shared interest where you can work on together. So, meet each other, and create a vision [on collaboration] and put your energy into it, that is effective.” (General practitioner, involved in a collaborative initiative on early detection)

Interviewees further indicated that a *shared vision* on early detection promotes collaboration. Respondents from the group of ‘other stakeholders’ in particular referred to the importance of a shared vision on early detection. According to them, professionals (e.g. general practitioners and social care professionals) have different views on early detection and on strategies for case finding. They use for instance different instruments for frailty screening or target their initiative on different subgroups of older people. These differences hamper collaboration. Stakeholders from the group ‘service providers’ particularly referred to a shared vision on setting up a collaborative initiative on early detection. What are, for instance, shared motivations and what can be the benefits of collaboration? The respondents indicated that a shared vision will support professionals to align their work and to coordinate their activities accordingly.

According to some interviewees, particularly service providers and representatives of municipalities, another facilitating factor is having seen *the benefits of collaboration*. A benefit is, for example, a decrease in the number of general practitioner visits by older adults with social care needs (e.g. loneliness), because such problems are already being detected and dealt with by a local team of social workers. These types of benefits were considered to positively affect willingness of professionals to their maintain collaboration.

In addition, *available time* was, with the exception of health insurers, mentioned by all respondents as an important factor affecting collaboration. Respondents, however, had different views on the factor ‘time’. Service providers for instance particularly mentioned that preventive activities, such as early detection, take time. Several professionals, such as general practitioners, are often busy with their daily duties and therefore have to be selective in any additional activities. Moreover, general practitioners tend to focus more on curative than on preventive activities, and are therefore less likely to practice such preventive activities or show a willingness to take part in these activities. Other respondents, such as municipality policymakers and academics indicated that setting up a collaborative initiative requires time, e.g. time required for alignment between all stakeholders or time required for motivating stakeholders. Not only early detection but also maintaining the organisational structures for collaboration are additional tasks to the other tasks professionals in health and social care already have. Taking up any additional responsibilities, on top of one’s daily duties, is for some professionals a barrier for collaboration.

Service providers, academics and representatives of older people, mentioned the *lack of a single shared patient information system* as a barrier to collaboration. Patient information systems often differ between organizations involved in early detection of frailty. This implies that health and social care professionals cannot electronically share information about their clients, such as outcomes of needs assessments, care plans, and delivery of care and support. This means that health and social care professionals often do not inform each other, cannot access each other’s information and do not discuss who will take care of which older adult.

The *organizational structure of municipalities* was mentioned by almost all interviewees as a barrier for collaboration. However, the impact that the interviewees mentioned, differed. Managers and health insurers noted that the different strategies for early detection of the different departments within municipalities hampered the collaboration. Other respondents, such as health and social care professionals, policymakers and representatives of older people, indicated that the fact that responsibility for preventive activities, such as early detection, are spread across different departments within municipalities (e.g. social care, housing, sports) hinders collaboration. Also, employees of municipalities are not always aware of each other’s activities and therefore do not know which initiatives are implemented in which neighbourhoods. Consequently, also health and social care professionals within the neighbourhoods do not collaborate resulting in duplicate tasks and activities.

“Moreover, neighbourhood information officer, also volunteers, who work on behalf of the municipality, well, they [municipalities policymakers] don’t know which information they collect and what happens with the information. Now there are different district counsellors working in the same neighbourhood. This is not working. At least alignment of what is happening could be improved.” (Social care professional, involved in a collaborative initiative on early detection)

With the exception of older people’s representatives, all respondents indicated that *fragmented funding* of health and social care was a barrier. The interviews showed, however, that professionals have different strategies for dealing with fragmented funding. Some respondents indicated that fragmented funding is a major barrier, making collaboration hardly possible. Other respondents indicated that, even though this is a barrier, fragmented funding should not be a reason for not collaborating.

“We really like to change fragmentation and we would really like to have bundled payment for health and social care. […] There is some progress, in the meantime. However, we do not have a real system change, because we have to deal with national laws and regulations, but meanwhile we work our way around it [laws and regulations].” (Municipality policymaker)

“Well, a barrier, you can work around it, but it is a barrier. And then you just need to have the right person within a health insurance company who says: “We give it a go” and the same holds of course for the municipality, that the person there also says “We give it a go” […]. What you need is a number of agencies that are motivated and that dare to take a risk. However, due to the fragmented funding, people like to stay safe using their own budgets and because of that, collaboration is never really taking off. “(Municipality policymaker)

### Cultural and/or professional level

A factor on the cultural and professional level hindering collaboration is a *lack of knowledge*. Several interviewees, such as service providers and commissioners, noted that health and social care professionals are still unsure about what would be the best strategy for early detection of frailty (see Table 2). They have questions such as ‘what types of instruments should be used for frailty screening and needs assessments?’ and ‘which health and/or social care professionals should be detecting what, and in which group of older people?’ These uncertainties also lead to uncertainties about who would be the best collaboration partners.

There is also a *lack of knowledge of privacy legislations*, which is hampering collaboration. Almost all respondents mentioned that people involved in early detection, such as health and social care professionals, volunteers, and informal carers often do not know which signals they are allowed to pass on to whom. For example, volunteers and informal carers sometimes falsely think that information about problems that they observe, such as loneliness or health problems, may not be passed on to health and social care professionals. This assumption is a barrier for collaboration with and between volunteers, informal carers and health and social care professionals.

Stakeholders from all three groups mentioned that *mutual trust* is of major importance. Having trust in other organizations’ knowledge and skills facilitates collaboration. Several respondents wondered whether there was sufficient trust in their way of working and judgement. They mentioned for instance that, although case managers from their care organizations already did a needs assessment, municipalities nevertheless decided that a social care consultant from their municipality also had to complete an assessment. They not only perceived these duplicate activities as a lack of trust, but also found them inefficient and unnecessary.

“What is going well in our collaboration is that we trusted each other quite quickly at different levels of our organizations Also, we discussed things that did not go well quite easily at the highest level […] So, that is a really important factor for success, organizations trusting each other’s way of working.” (Representative of health insurers, involved in a collaborative initiative on early detection)

In addition, a *lack of leadership* and *ownership* was described as a barrier for collaboration. This was mentioned by all groups of respondents. Interviewees described that agreements regarding collaboration with, for example health insurers, municipalities and network organisations did not result into action, since no one felt ownership. They noted that ambassadors could play a stimulating role in setting up and developing collaboration programs. These ambassadors can for example be inspiring care professionals or public health advisors. They could initiate or speed up collaboration processes, could connect professionals or play a coordinating role.

“Well, yes, we have two ambassadors. If we would not have them, then it would have been hard to stay focused. It is hard to keep everyone involved, then it will be very, very difficult and then you’ll see processes are going slower.” (Municipality policymaker)

“Yes, what makes it difficult is that you still want to give a lot of responsibility to the people who work in the field, despite the fact that we are financing it [early detection initiatives]. We do hand over responsibilities but these are nevertheless not always taken. Regularly we hear: “This goes wrong and that goes wrong and that has to be different”. Our response is then: how are we going to solve this? And sometimes, even when there are financial resources, we don’t get an answer. So, yes, when there is ownership, people feel responsible for a problem, but we also need some taking ownership for a solution.” (Municipality policymaker)

### Contextual level

Almost all interviewees mentioned that *limited and/or temporary funding for collaboration* is a barrier (see Table 2). Establishing collaborations and collaborating in the complex field of early detection take time and resources. Some aspects of collaborative initiatives on early detection, such as multidisciplinary meetings concerning older adults with complex needs, require professionals to invest additional time, on top of their daily duties. Often, there is only limited budget for such activities, making it difficult for professionals to justify their attendance at those meetings. Also, consultation of particular professionals, such as elderly care specialists for medication review, takes time and resources, which is often not covered by funding.

“Funding is a disaster, but it differs. Nationwide we see for instance that a lot of health insurers differ in the extent to which they are willing to invest in activities that go beyond those related to individual treatments. […]. Municipalities and health insurers are assigned to collaborate in the somewhat ‘blurred’ area of prevention. However, there are only few health insurers that are actually doing this. I think, they are very reluctant to do so, and certainly on the intersection of health and social care.” (Manager of a social care organization)

“Well, I think it is really difficult to put things in motion […] And, in my opinion, that is related to how they [organizations] are funded. […] And it is still rather difficult to work on smart local alliances, especially in addition to your core businesses.” (Municipality policymaker)

Another contextual barrier mentioned by interviewees from all three groups is the *lack of continuity of professionals* they work with. Due to changes in collaboration partners, professionals continuously have to become acquainted with new partners and have to make new arrangements and agreements. Interviewees indicated that collaborations’ success often depend on individuals and not so much on their positions. It requires a time investment to get to know new collaboration partners.

“And it’s frustrating, I just mentioned staff changes in different organisations, the municipality, health insurers, you name it […] staff changes in your own team, are also very frustrating. In the past three years, I worked with three different elderly care specialists, four different community nurses, which is also quite tiring.” (Health care professional, involved in a collaborative initiative on early detection)

## Discussion

### Main findings

In response to the increasing number of older people, countries are developing strategies to optimally support older people in their home environments, one of which is proactively detecting frailty and initiating appropriate interventions addressing these needs. Since older people often face multiple health and social care needs [23], often multiple health and social care providers and agencies are involved in delivering care and support to them [17,19]. The first aim of our study was to provide insight into the factors that impede or facilitate collaboration between the different actors involved in early detection initiatives. This study shows, in line with previous studies [19, 24,25,26,27,28,29,30], that inter-sectoral collaboration is challenging, and that multiple factors play a role in whether or not collaborations are established or are successful. Some of these factors seem to be supportive for successful collaboration, such as knowing each other and everyone’s expertise and mutual trust, whereas others seem to be unsupportive, such as a lacking a shared patient information system or a common financing system. In general, the factors perceived to influence collaboration on early detection were not different from the factors mentioned in other international studies on integrated care [21,24,27], that impact the collaboration between health and social care providers in other areas. For instance, developing a vision shared by all service providers involved at a local level, is generally considered key for successful collaboration. However, in the case of early detection of frailty, this may be (even) more complicated due to the ambiguity of the frailty concept and the lack of knowledge on effective detection approaches.

As in previous studies, the factors identified in this study were related to different levels of the health and care system. These include the contextual level (e.g. limited funding for collaboration), organizational level (e.g. shared vision, shared information system, available time, awareness of one another’s roles and competences), and cultural and professional level (e.g. mutual trust, leadership, having a collaboration champion or facilitator). There are interdependencies among and between factors at all three levels. The availability of funding will for instance determine the amount of time the different actors spend on the collaboration, whereas knowledge of each other’s tasks and responsibilities will probably increase mutual trust. The Rainbow Model of Integrated Care [31,32] shows how collaboration-supportive policies and actions at the contextual level (e.g. designing a collaboration-supportive financing system) could facilitate collaboration at the organizational and professional level. This also holds for developing a shared vision as part of inter-sectoral or inter-organizational collaboration, which will facilitate inter-professional collaboration [31,32]. Although, no studies specifically on collaboration on early detection of frailty on other counties were found, barriers such as the lack of coordination between different policy levels and the challenge of the efficient sharing of information have come to the fore as barriers of inter-organisational and inter-sectoral collaboration in other countries as well [33].

The second aim of our study was to gain insight in whether different groups of actors, such as commissioners, managers and professionals of care organisations, advisors, academics and representatives of organizations for older people, hold different views regarding facilitators and barriers to detect frailty among older citizens. Overall, the different actors participating in our study mentioned similar factors. Some differences may be worth noting, although we should acknowledge that sample sizes of some of the groups of actors were small. Health insurers and policymakers of municipalities for instance did not mention some of the factors that were important for service providers, such as the importance of a shared information system. Health insurers additionally mentioned fewer barriers than the other groups of actors. An obvious explanation for these findings is that policymakers and health insurers, as commissioners of early detection, are not involved in the daily practice of early detection and therefore do not face similar issues as service providers. It should further be noted that, particularly health and social care providers and representatives of organizations for older people, mentioned the uncertainty of professionals about what would be the best strategy for early detection of frailty. Consequently, they were also unsure about who would be the best collaboration partners regarding early detection and therefore had difficulties to initiate a collaboration. This uncertainty is also reflected by other studies. Some systematic reviews for instance show that recognizing (potential) frailty at an early stage could delay or reduce functional decline, prevent hospital admissions, and maintain wellbeing and independent living for as long as possible [9,10,11,12]. Other reviews do, however, not underline such outcomes [14,34,35]. This illustrates that in practice as well as in science, there are various views on early detection and its potential benefits. Although the different actors in general mentioned similar factors, they tended to have different views and experiences with the impact of these factors. For example, the factor ‘fragmented funding’ was mentioned by almost all interviewees. Some actors mentioned that fragmented funding was a major barrier, making collaboration hardly possible. Other respondents, however, indicated that, although a lack of funding is a barrier, this should not be a reason for not collaborating.

### Methodological considerations

For this study, we interviewed a wide range of actors in proactive elderly care in the Netherlands, including commissioners, different types of service providers (both managers and care professionals), and other types of stakeholders (i.e. advisors, academics, representatives of organizations for older people). Some actors were directly involved in a collaborative initiative on early detection. Others were not directly involved in a collaborative initiative but had a visionary point of view on proactive elderly care. This variety of participants is a strength of this study, since it resulted in a broad perspective regarding the facilitators and barriers for collaboration between actors involved in early detection. At the same time, however, we need to acknowledge that the number of respondents per group of actors was small. This was particularly the case for the health insurers, academics, and representatives of organizations for older people. To overcome this limitation, we aimed to validate answers given by these actors for as much as possible in the other groups of actors. This was to verify whether similar barriers and facilitators were experienced by other actors, and to verify whether these groups of actors were familiar with these factors without necessarily experiencing them themselves.

By applying qualitative research methods and analysing data from different perspectives, we aimed to gain a deeper understanding of the factors that underlie (a lack of) collaboration between professionals and agencies involved in early detection of frailty among older persons. We did not establish which issues occurred more frequently than others or to compare different groups of actors to detect potential statistical differences. To answer these types of questions, future studies that also apply quantitative methods is recommended.

### Implications for policy and practice

Our study suggests that different actors involved in early detection seem to experience similar facilitators and barriers for collaboration although their views on these factors may sometimes differ. These insights help to define the required next steps for improving collaboration between professionals and agencies involved in early detection at a local level. Potential solutions focus on different stakeholders, both at the local and national level. Therefore, we have formulated recommendations for both service providers and policymakers and actors at national level.

Based on our study, service providers are recommended to actively contact relevant organizations in their neighbourhoods or regions. Becoming familiar with local organizations in the field of early detection and getting to know each other is a first step to establish collaboration. It is important to discuss each other’s roles and responsibilities and to get clarity on where ones’ roles and responsibilities end and where those of others start. It is possible that the different policy levels define and/or limit roles and responsibility of actors. Is should be considered that there is broad legal basis for early detection providing different approaches to early detection, including various levels of accountability and execution. Service providers are further recommended to develop a shared vision on what organisations want to achieve with early detection and how local collaboration could contribute to this vision. It is important to look for common ground when discussing this vision. To create further commitment within and between organisations, it is recommended to involve professionals from various levels within organisations, that include both health and social care professionals as well as managers, in creating a vision. This might also help to resolve some of the insecurities health and social care professionals have regarding their strategy for early detection.

In line with other studies [21,26,27], we further recommend service providers to explore possibilities for a shared patient/client information system. This study shows that a shared patient/client information system is useful for efficient collaboration. Sharing information will help to prevent duplicate activities or activities not being done at all. It is also important to take the General Data Protection Regulation (GDPR) into account [36], while working with personal data. An ongoing study on privacy-aware and acceptable lifelogging services for older and frail people might provide useful information on sharing information and privacy matters [37]. Another recommendation is to consider how collaborations can be imbedded in existing networks and collaborations. This is to prevent multiple collaboration structures within one municipality or region. A final recommendation for service providers is to share knowledge about good practices. In the Netherlands, but also in other countries, there are several examples of good practices in collaboration in early detection [38,39]. Learning from these initiatives (e.g. how to give shape to a collaboration, how to share information, how to create a shared vision) will help to prevent ‘reinventing the wheel’.

Recommendations for policymakers and actors at national level include the integration of inter-professional collaboration in frailty education and training of health and social care professionals. This might help to create trust, to identify shared interests and to learn to speak each other’s language early on. It is further recommended, since early detection initiatives are now being funded from different budgets, to explore possibilities to overcome the experienced financial barriers. Potential solutions may be more collaboration between the commissioners of early detection, i.e. municipalities and health insurers, and exploring possibilities of bundled payments.

## Conclusion

Collaborating on early detection of frailty appears to be a multifaceted challenge. The facilitators and barriers at the organizational level are knowing each other’s tasks and responsibilities, shared vision on early detection, saving seen the benefits of collaboration, time available, shared patient information system, organizational structure of municipalities and the way in which professionals deal with fragmented funding. The facilitators and barriers at the cultural and/or professional level are knowledge on early detection approaches, knowledge on privacy legislation, trust and leadership. The contextual facilitators and barriers are funding for collaboration and continuity of cooperation partners. However, as different stakeholders hold similar views, there seem to be several starting-points to improve collaboration. First steps shall include getting to know each other and developing a shared vision on early detection.

## References

[1] Cesari, M, et al. Frailty: An emerging public health priority. Journal of the American Medical Directors Association, 2016; 17(3): 188–192. DOI: 10.1016/j.jamda.2015.12.016 26805753

[2] Collard, RM, et al. Prevalence of frailty in community dwelling older persons: A systematic review. Journal of the American Geriatrics Society, 2012; 60(8): 1487–1492. DOI: 10.1111/j.1532-5415.2012.04054.x 22881367

[3] Markle Reid, M and Browne, G. Conceptualizations of frailty in relation to older adults. Journal of Advanced Nursing, 2003; 44(1): 58–68. DOI: 10.1046/j.1365-2648.2003.02767.x 12956670

[4] O’Caoimh, R, et al. Frailty at population level: A systematic review. Report ADVANTAGE Joint Action; 2017. 10.4415/ANN_18_03_10 30284550

[5] Fried, LP, et al. Untangling the concepts of disability, frailty, and comorbidity: Implications for improved targeting and care. The Journals of Gerontology Series A: Biological Sciences and Medical Sciences, 2004; 59(3): 255–263. DOI: 10.1093/gerona/59.3.M255 15031310

[6] Vermeiren, S, et al. Frailty and the prediction of negative health outcomes: A meta-analysis. Journal of the American Medical Directors Association, 2016; 17(12): 1163.e1–1163.e17. DOI: 10.1016/j.jamda.2016.09.010 27886869

[7] Lahousse, L, et al. Adverse outcomes of frailty in the elderly: The Rotterdam Study. European Journal of Epidemiology, 2014; 29(6): 419–427. DOI: 10.1007/s10654-014-9924-1 24935872

[8] Hoogendijk, EO, et al. Adverse effects of frailty on social functioning in older adults: Results from the Longitudinal Aging Study Amsterdam. Maturitas, 2016; 83: 45–50. DOI: 10.1016/j.maturitas.2015.09.002 26428078

[9] Stuck, AE, et al. Home visits to prevent nursing home admission and functional decline in elderly people: Systematic review and meta-regression analysis. Journal of the American Medical Association, 2002; 287(8): 1022–1028. DOI: 10.1001/jama.287.8.1022 11866651

[10] Beswick, AD, et al. Complex interventions to improve physical function and maintain independent living in elderly people: A systematic review and meta-analysis. The Lancet, 2008; 371(9614): 725–735. DOI: 10.1016/S0140-6736(08)60342-6 PMC2262920 18313501

[11] Huss, A, et al. Multidimensional preventive home visit programs for community-dwelling older adults: A systematic review and meta-analysis of randomized controlled trials. The Journals of Gerontology. Series A, Biological Sciences and Medical Sciences, 2008; 63(3): 298–307. DOI: 10.1093/gerona/63.3.298 18375879

[12] Elkan, R, et al. Effectiveness of home based support for older people: Systematic review and meta-analysisCommentary: When, where, and why do preventive home visits work? British Medical Journal, 2001; 323(7315): 719 DOI: 10.1136/bmj.323.7315.719 11576978 PMC56889

[13] Dury, S, et al. Detecting frail, older adults and identifying their strengths: Results of a mixed-methods study. BMC Public Health, 2018; 18(1): 191 DOI: 10.1186/s12889-018-5088-3 29378540 PMC5789734

[14] Mayo-Wilson, E, et al. Preventive home visits for mortality, morbidity, and institutionalization in older adults: A systematic review and meta-analysis. PLOS ONE, 2014; 9(3): e89257 DOI: 10.1371/journal.pone.0089257 24622676 PMC3951196

[15] Counsell, SR, et al. Geriatric care management for low-income seniors: A randomized controlled trial. JAMA, 2007; 298(22): 2623–2633. DOI: 10.1001/jama.298.22.2623 18073358

[16] Van Kempen, J. The identification of frail older persons in primary care: The development and validation of the EASY-Care Two step Older persons screening; 2013 Radboud University Nijmegen.

[17] Lette, M, et al. Initiatives on early detection and intervention to proactively identify health and social problems in older people: Experiences from the Netherlands. BMC Geriatrics, 2015; 15(1): 143 DOI: 10.1186/s12877-015-0131-z 26518369 PMC4628317

[18] Bleijenberg, N, et al. Development of a proactive care program (U-CARE) to preserve physical functioning of frail older people in primary care. Journal of Nursing Scholarship, 2013; 45(3): 230–237. DOI: 10.1111/jnu.12023 23530956

[19] Lette, M, et al. Improving early detection initiatives: A qualitative study exploring perspectives of older people and professionals. BMC Geriatrics, 2017; 17(1): 132 DOI: 10.1186/s12877-017-0521-5 28645251 PMC5482941

[20] Gale, NK, et al. Using the framework method for the analysis of qualitative data in multi-disciplinary health research. BMC Medical Research Methodology, 2013; 13: 117 DOI: 10.1186/1471-2288-13-117 24047204 PMC3848812

[21] Cameron, A, et al. Factors that promote and hinder joint and integrated working between health and social care services: A review of research literature. Health & Social Care in the Community, 2014; 22(3): 225–233. DOI: 10.1111/hsc.12057 23750908

[22] Giacomini, MK, Cook, DJ and E-BMW Group. Users’ guides to the medical literature: XXIII. Qualitative research in health care A. Are the results of the study valid? Journal of the American Medical Association, 2000; 284(3): 357–362. DOI: 10.1001/jama.284.3.357 10891968

[23] Michel, JP, et al. Oxford Textbook of Geriatric Medicine, 2018; 429 Oxford: Oxford University Press.

[24] Karam, M, et al. Comparing interprofessional and interorganizational collaboration in healthcare: A systematic review of the qualitative research. International Journal of Nursing Studies, 2018; 79: 70–83. DOI: 10.1016/j.ijnurstu.2017.11.002 29202313

[25] Supper, I, et al. Interprofessional collaboration in primary health care: A review of facilitators and barriers perceived by involved actors. Journal of Public Health, 2015; 37(4): 716–727. DOI: 10.1093/pubmed/fdu102 25525194

[26] Mulvale, G, Embrett, M and Razavi, SD. ‘Gearing Up’to improve interprofessional collaboration in primary care: A systematic review and conceptual framework. BMC Family Practice, 2016; 17(1): 83 DOI: 10.1186/s12875-016-0492-1 27440181 PMC4955241

[27] Van Dongen, JJJ, et al. Interprofessional collaboration regarding patients’ care plans in primary care: A focus group study into influential factors. BMC Family Practice, 2016; 17(1): 58 DOI: 10.1186/s12875-016-0456-5 27233362 PMC4884411

[28] Xyrichis, A and Lowton, K. What fosters or prevents interprofessional teamworking in primary and community care? A literature review. International Journal of Nursing Studies, 2008; 45(1): 140–153. DOI: 10.1016/j.ijnurstu.2007.01.015 17383655

[29] Poulton, BC and West, MA. The determinants of effectiveness in primary health care teams. Journal of Interprofessional Care, 1999; 13(1): 7–18. DOI: 10.3109/13561829909025531

[30] Körner, M, et al. Interprofessional teamwork and team interventions in chronic care: A systematic review. Journal of Interprofessional Care, 2016; 30(1): 15–28. DOI: 10.3109/13561820.2015.1051616 26709985

[31] Valentijn, PP, et al. Understanding integrated care: A comprehensive conceptual framework based on the integrative functions of primary care. International Journal of Integrated Care, 2013; 13 DOI: 10.5334/ijic.886 PMC3653278 23687482

[32] Valentijn, PP. Rainbow of chaos: A study into the theory and practice of integrated primary care. International Journal of Integrated Care, 2016; 16(2). DOI: 10.5334/ijic.2465

[33] Hujala, A, Taskinen, H and Rissanen, S. How to support integration to promote care for people with multimorbidity in Europe? World Health Organization, Regional Office for Europe; 2017. 29144694

[34] Van Haastregt, JC, et al. Effects of preventive home visits to elderly people living in the community: Systematic review. British Medical Journal, 2000; 320(7237): 754–758. DOI: 10.1136/bmj.320.7237.754 10720360 PMC27318

[35] Bouman, A, et al. Effects of intensive home visiting programs for older people with poor health status: A systematic review. BMC Health Services Research, 2008; 8(1): 74 DOI: 10.1186/1472-6963-8-74 18387184 PMC2364620

[36] European Commision. 2018 reform of EU data protection rules; 2018.

[37] Florez-Revuelta, F, et al. Privacy-Aware and Acceptable Lifelogging services for older and frail people: The PAAL project. In: 2018 IEEE 8th International Conference on Consumer Electronics-Berlin (ICCE-Berlin); 2018 IEEE DOI: 10.1109/ICCE-Berlin.2018.8576191

[38] Bleijenberg, N, et al. Exploring the expectations, needs and experiences of general practitioners and nurses towards a proactive and structured care programme for frail older patients: A mixed-methods study. Journal of Advanced Nursing, 2013; 69(10): 2262–2273. DOI: 10.1111/jan.12110 23461433

[39] Spoorenberg, SL, et al. Embrace, a model for integrated elderly care: Study protocol of a randomized controlled trial on the effectiveness regarding patient outcomes, service use, costs, and quality of care. BMC Geriatrics, 2013; 13(1): 62 DOI: 10.1186/1471-2318-13-62 23782932 PMC3702391

